# Prokaryotic expression and characterization of the heterodimeric construction of ZnT8 and its application for autoantibodies detection in diabetes mellitus

**DOI:** 10.1186/s12934-017-0816-4

**Published:** 2017-11-13

**Authors:** Natalia I. Faccinetti, Luciano L. Guerra, Adriana V. Sabljic, Silvina S. Bombicino, Bruno D. Rovitto, Ruben F. Iacono, Edgardo Poskus, Aldana Trabucchi, Silvina N. Valdez

**Affiliations:** 1 Universidad de Buenos Aires, Facultad de Farmacia y Bioquímica, Departamento de Microbiología, Inmunología, Biotecnología y Genética, Cátedra de Inmunología, Buenos Aires, Argentina; 2 CONICET-Universidad de Buenos Aires, Instituto de Estudios de la Inmunidad Humoral “Prof. Ricardo A. Margni” (IDEHU), Buenos Aires, Argentina

**Keywords:** Diabetes mellitus, ZnT8, Autoantibody, Recombinant protein expression, Immunoassays, *Escherichia coli*

## Abstract

**Background:**

In the present work we described the recombinant production and characterization of heterodimeric construction ZnT8-Arg-Trp325 fused to thioredoxin using a high-performance expression system such as *Escherichia coli*. In addition, we apply this novel recombinant antigen in a non-radiometric method, with high sensitivity, low operational complexity and lower costs.

**Results:**

ZnT8 was expressed in *E. coli* as a fusion protein with thioredoxin (TrxZnT8). After 3 h for induction, recombinant protein was obtained from the intracellular soluble fraction and from inclusion bodies and purified by affinity chromatography. The expression and purification steps, analyzed by SDS-PAGE and western blot, revealed a band compatible with TrxZnT8 expected theoretical molecular weight (≈ 36.8 kDa). The immunochemical ability of TrxZnT8 to compete with [^35^S]ZnT8 (synthesized with rabbit reticulocyte lysate system) was assessed qualitatively by incubating ZnT8A positive patient sera in the presence of 0.2–0.3 μM TrxZnT8. Results were expressed as standard deviation scores (SDs). All sera became virtually negative under antigen excess (19.26–1.29 for TrxZnT8). Also, radiometric quantitative competition assays with ZnT8A positive patient sera were performed by adding TrxZnT8 (37.0 pM–2.2 µM), using [^35^S]ZnT8. All dose–response curves showed similar protein concentration that caused 50% inhibition (14.9–0.15 nM for TrxZnT8). On the other hand, preincubated bridge ELISA for ZnT8A detection was developed. This assay showed 51.7% of sensitivity and 97.1% of specificity.

**Conclusions:**

It was possible to obtain with high-yield purified heterodimeric construction of ZnT8 in *E. coli* and it was applied in cost-effective immunoassay for ZnT8A detection.

**Electronic supplementary material:**

The online version of this article (10.1186/s12934-017-0816-4) contains supplementary material, which is available to authorized users.

## Background

Zinc transporter 8 (ZnT8) is a multipass transmembrane protein which has been identified as a novel autoantigen in patients with diabetes mellitus (DM), such as in type 1 DM and in latent autoimmune diabetes of the adults (LADA) [1, 2]. It is a 369 amino acid protein, encoded by the SLC30A8 gene located in the chromosome 8q24.11. ZnT8 transports zinc ions from the beta-cell cytoplasm into insulin-secretory vesicles where they are essential for the proper storage and secretion of insulin [3]. Genome-wide association studies analyzing susceptible/protective loci for type 2 DM revealed that a nonsynonymous single nucleotide polymorphism (SNP) in SLC30A8 (rs13266634 C>T) is associated with the disease [4]. This SNP changes arginine (Arg) to tryptophan (Trp) at position 325, with a higher risk of developing the disease when the allele C (Arg325) is present.

Among the major autoantibodies employed in the diagnosis of autoimmune DM, autoantibodies to ZnT8 (ZnT8A) is the most recently described humoral marker, complementing those already used such as insulin/proinsulin autoantibodies (IAA/PAA), glutamic acid decarboxylase autoantibodies (GADA), and insulinoma associated protein tyrosine phosphatase 2 autoantibodies (IA-2A).

Wenzlau et al. and other authors have reported that ZnT8-reactive sera mostly recognize the C-terminal domain of the molecule (amino acids 268–369) [1, 5]. ZnT8A have been detected in more than 60% of patients with type 1 DM [1, 2, 6–11] and in more than 10% of adult-onset diabetic patients [2, 8, 12], increasing the sensitivity in the screening of autoimmunity. Therefore, ZnT8A constitutes an additional prevalent marker to the preexisting triad, enabling the appropriate classification of diabetic patients as autoimmune DM and improving the efficacy of treatment.

The reference method for detection of ZnT8A is the radioligand binding assay (RBA); the antibody standardization program (DASP) demonstrated that this method achieved high sensitivity and specificity [13]. However, this assay includes a recombinant radiolabeled autoantigen produced by cell-free protein expression systems, which is environmentally inappropriate, expensive to produce or unsustainable for wide application in laboratories or clinical settings.

We have previously characterized ZnT8A from type 1 diabetic patients and non-obese adult-onset diabetic patients employing different ZnT8 antigenic variants, since the C-terminal domain of the ZnT8 include the variant residue at amino acid 325. In that work, we concluded that the heterodimeric construction ZnT8-Arg-Trp325 showed higher prevalence and signal levels and increased dynamic range in RBA for ZnT8A detection in both group of patients, therefore we decided to continue working with this antigenic construction [2]. Although there are commercial ELISA kits for ZnT8A detection, most of them do not mention their specificity and sensitivity parameters. In particular, ELISA RSR™ZnT8 Ab™ kit reports 97% specificity and 76% of sensitivity in IASP 2015 (RSR Limited, UK). However, none of these kits indicate the ZnT8 construction employed.

Given the need for improved and less expensive ZnT8A assays, in the present work we described the recombinant production and characterization of the heterodimeric construction ZnT8-Arg-Trp325 fused to thioredoxin (Trx) using a high-performance expression system such as *Escherichia coli*. Additionally, in this work, we apply this novel recombinant antigen in a non-radiometric method, with high sensitivity, low operational complexity and lower costs such as ELISA.

## Results

### Expression of ZnT8 as a fusion protein with thioredoxin in *E. coli*

Recombinant TrxZnT8 was expressed in both strains of *E. coli*, GI698 and GI724. Efficient TrxZnT8 production was achieved after 3 h of protein expression induction in both strains. Western blot (WB) analysis of intracellular soluble fraction (ISF) and inclusion bodies (IB) with polyclonal serum to Trx showed one band with the expected molecular weight (MW, ≈ 36.8 kDa) for the full-length engineered protein, whereas such bands were absent in the ISF and IB from non-transformed bacteria under the same experimental conditions (Additional file 1: Figure S1). The highest levels of protein expression were obtained after 3 h of induction in ISF from strain GI698 and in IB from strain GI724.

### TrxZnT8 from the intracellular soluble fraction of *E. coli* GI698

#### Purification of TrxZnT8 from the intracellular soluble fraction

**Fig. 1:**
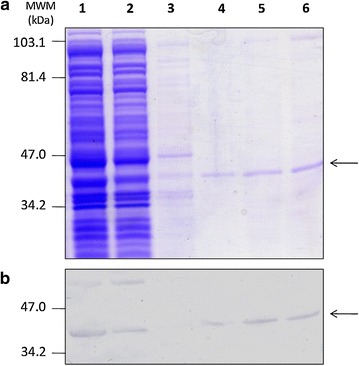
Purification of TrxZnT8 from ISF by affinity chromatography. Analysis of TrxZnT8 fractions at different stages of purification by **a** SDS-PAGE (12.1% T, 6.0% C, 1 mm, under reducing conditions, stained with Coomassie Brilliant Blue R-250) and **b** WB revealed with a rabbit polyclonal serum to thioredoxin as primary antibody. In **a** and **b**, lane 1 corresponds to total ISF from transformed *E. coli* strain GI698, lane 2: unbound material, lane 3 and 4: washes with increasing 2ME concentrations (1 and 5 mM), lanes 5 and 6: 100 mM consecutive eluates of purified TrxZnT8. Arrows indicate the electrophoretic mobility of TrxZnT8

Recombinant TrxZnT8 was purified from the ISF of *E. coli* strain GI698 after 3 h of induction. The procedure was based on an affinity chromatography in which the Trx portion of TrxZnT8 binds to the resin by its catalytic domain containing vicinal dithiols. This one-step purification separated most contaminant proteins with little or no significant loss of TrxZnT8 relying on the high capacity of the in-house made resin. Figure 1 depicts SDS-PAGE and WB of different stages of purification. Based on the quantification of the 100 mM 2ME fraction (lanes 5–6) bands, the purification yielded ≈ 1.25 mg of 72.0–74.0% pure TrxZnT8/L of culture medium.

#### Biochemical characterization of TrxZnT8

In order to estimate the MW of TrxZnT8, an SDS-PAGE with MW calibrators (bovine albumin, ovalbumin, carbonic anhydrase and trypsin inhibitor) was performed. A calibration curve of log MW vs. R_f_ was constructed (log MW = − 0.0806 × R_f_ + 5.071, R^2^ = 0.9960) and the unknown MW of TrxZnT8 was interpolated. An experimental MW (38,474 Da) compatible to the theoretical MW (36,847 Da) was obtained (4.4% error, which is a satisfactory accuracy for this method) [14].

**Fig. 2:**
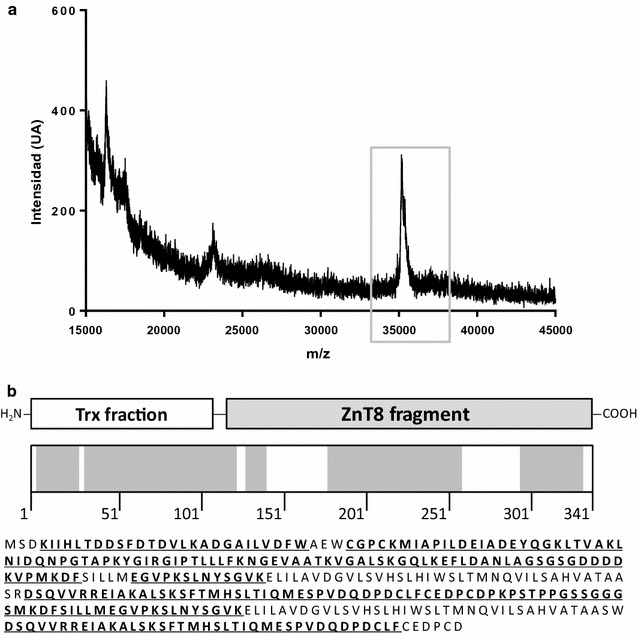
Biochemical characterization of TrxZnT8. **a** Mass spectrometry analysis of recombinant TrxZnT8. The box indicates the experimental peak corresponding to TrxZnT8. **b** TrxZnT8 scheme (above) and sequence (below). In the scheme, N-terminal Trx fraction, the linker peptide and the ZnT8 fragment in the C-terminal of the construction are detailed, and the corresponding peptides fragments identified by Orbitrap Mass Spectrometry are highlighted in grey in the diagram. Also, the TrxZnT8 sequence is represented: underlined and highlighted in bold are those fragments identified by Orbitrap Mass Spectrometry considering high confidence peptides

Mass spectrometry analysis of TrxZnT8 verified the agreement of the MW of our expressed chimera with the expected value (actual mass 37,042.770 Da; theoretical mass 36,847.200 Da) (Fig. 2a). Besides, purified TrxZnT8 was also subjected to proteolytic digestion with trypsin or chymotrypsin. The peptide profile obtained after each digestion was analyzed by Orbitrap mass spectrometry, achieving 73.6% total coverage (34.3% for trypsin and 62.7% for chymotrypsin) of TrxZnT8 (341 residues) (Fig. 2b), considering high confidence peptides only; when low confidence peptides were not filtered, 91.50% coverage of the theoretical composition of the protein was accounted for.

#### Immunochemical characterization of TrxZnT8

**Fig. 3:**
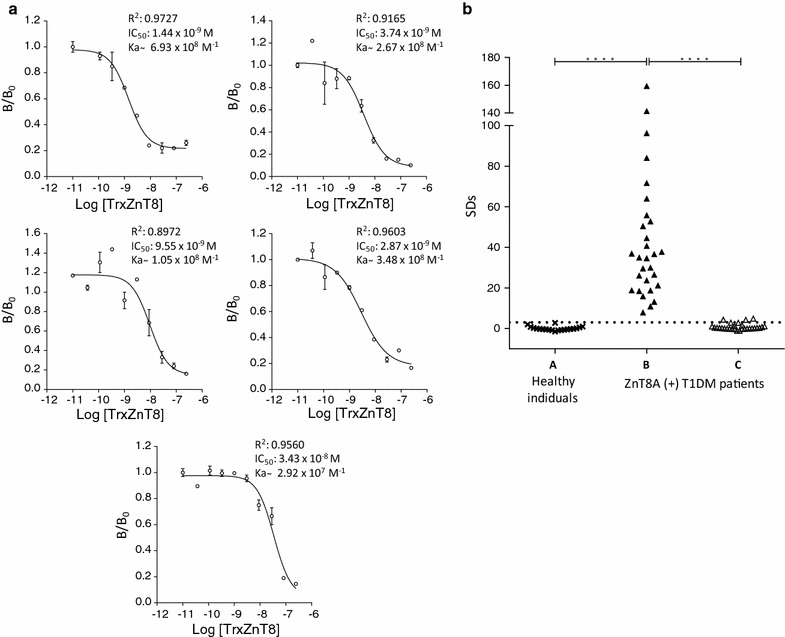
Immunochemical characterization of TrxZnT8 from ISF. **a** Dose–response curves for 5 ZnT8A RBA positive type 1 diabetic patient sera incubated with different concentrations of TrxZnT8. Each curve includes best-fit values from the log (inhibitor) vs. response–variable slope (four parameters) equation: correlation coefficient data (R^2^), IC_50_ values and approximation to the affinity constant of ZnT8A (K_a_) calculated as the inverse of IC_50_. **b** Inhibition capacity of TrxZnT8 assessed in 29 ZnT8A RBA positive type 1 diabetic patient sera in the absence (B) or presence (C) of TrxZnT8. Twenty control sera were used in order to set a cut-off value (A). The binding was expressed as SDs, and the dotted line represents the cut-off value

In order to test the ability of TrxZnT8 to react with ZnT8A, a quantitative competition assay was performed in which variable concentrations of TrxZnT8 were added (Fig. 3a). Five sera ZnT8A(+) from type 1 diabetic patients were used. The experimental data were fitted to the log [inhibitors] vs. response–variable slope (four parameters) equation using GraphPad Prism version 6.01. The TrxZnT8 concentration that caused 50% inhibition (IC_50_) was calculated for each serum. All dose–response curves showed similar IC_50_ (ranging from 9.55 × 10^−9^ to 3.43 × 10^−8^ M), indicating comparable TrxZnT8 immunoreactivity with ZnT8A.

In addition, inhibition assays were performed (Fig. 3b). Sera from 29 type 1 diabetic patients that scored positive by the reference method, RBA (with [^35^S]-ZnT8), were used [2, 12]. Also, 20 control sera were included in order to set a cut-off value. All patient sera tested were ZnT8A (+) with median SDs of 34.91, range 7.84–159.32 and cut-off value for positivity SDs = 3.0. A significant difference between sera from type 1 diabetic patients and healthy human individuals was observed (Unpaired samples Mann–Whitney *U* test, *p* < 0.0001). All type 1 diabetic patient sera became virtually negative under antigen excess: median SDs changed from 34.91 to 0.23 (range − 1.14 to 4.90) with TrxZnT8 excess (Wilcoxon test for paired samples, p < 0.0001).

### TrxZnT8 from inclusion bodies of *E. coli* GI724

#### Purification of TrxZnT8 from inclusion bodies

**Fig. 4:**
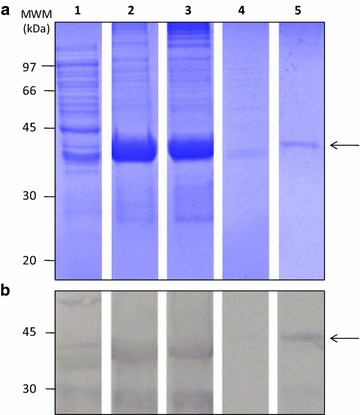
Purification of TrxZnT8 from IB by affinity chromatography. Analysis of TrxZnT8 fractions at different stages of purification by **a** SDS-PAGE (12.1% T, 6.0% C, 1 mm, under reducing conditions, stained with Coomassie Brillant Blue R-250) and **b** WB revealed with a rabbit polyclonal serum to thioredoxin as primary antibody. Lane 1 corresponds to total cell lysate from transformed *E.* *coli* strain GI724, lane 2 TrxZnT8 from IB solubilized with 8 M urea, lane 3 TrxZnT8 in oxidative refolding buffer, lane 4 unbound material, and lane 5 purified TrxZnT8 from IB. Arrows indicates the electrophoretic mobility of TrxZnT8

TrxZnT8 was also expressed in *E. coli* strain GI724 after 3 h of induction. The protein from IB was solubilized with 8 M urea in 0.1 M Tris, pH 8.5. After ON refolding, facilitated by a disulphide reduction–reoxidation procedure, TrxZnT8 was purified by affinity chromatography in which the Trx portion of TrxZnT8 was recognized by the anti-Trx antibodies present in the resin. Figure 4 depicts SDS-PAGE and WB of different fractions, from solubilization to purification. The one-step purification separated most contaminant proteins with little or no significant loss of TrxZnT8 relying on the high capacity of the in-house made resin. The purification yielded ≈ 2.20 mg of 95.0–97.0% pure TrxZnT8/L of culture medium.

#### Immunochemical characterization of TrxZnT8

**Fig. 5:**
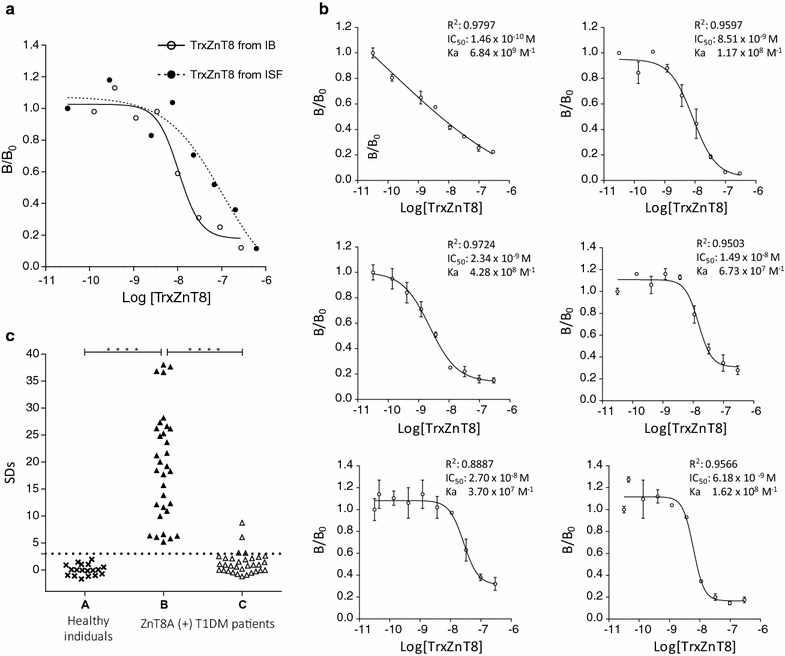
Immunochemical characterization of TrxZnT8 from IB. **a** Dose–response curves for a pool of 5 ZnT8A RBA positive type 1 diabetic patient sera for different concentrations of TrxZnT8 from ISF (closed circle, dotted line) and TrxZnT8 from IB (open circle, solid line). **b** Dose–response curves for six ZnT8A RBA positive type 1 diabetic patient sera incubated with different concentrations of TrxZnT8 from IB. Each curve includes best-fit values from the log (inhibitor) vs. response–variable slope (four parameters) equation: correlation coefficient data (R^2^), IC_50_ values and approximation to the affinity constant of ZnT8A (K_a_) calculated as the inverse of IC_50_. **c** Inhibition capacity of TrxZnT8 assessed in 33 ZnT8A RBA positive type 1 diabetic patient sera in the absence (B) or presence (C) of TrxZnT8. Twenty control sera were used in order to set a cut-off value (A). The binding was expressed as SDs, and the dotted line represents the cut-off value

Competitive quantitative and qualitative assays were also performed for TrxZnT8 from IB in order to evaluate any distortion in epitopes caused during the solubilization and refolding procedures. The immunochemical identities between TrxZnT8 from ISF and from IB were analyzed and expressed in terms of parallelism. As seen in Fig. 5a, when using a pool of ZnT8A + human sera, parallelism and identity between curves was achieved (one curve adequately fits all data, alpha = 0.05, R^2^ = 0.8796). Furthermore, quantitative competition assays were performed with six ZnT8A positive sera from type 1 diabetic patients (Fig. 5b). The experimental data were fitted to the log (inhibitors) vs. response–variable slope (four parameters) equation using GraphPad Prism version 6.01. The IC_50_ was calculated for each serum. All dose–response curves showed similar IC_50_ (ranging from 1.46 × 10^−10^ to 1.49 × 10^−8^ M), indicating comparable TrxZnT8 immunoreactivity with ZnT8A and similar to TrxZnT8 from ISF.

The inhibition assay for TrxZnT8 from IB was performed by incubating 33 type 1 diabetic patient sera in the presence of 290 nM TrxZnT8 (Fig. 5c). All tested patient sera were ZnT8A positive with a SDs of 19.26 ± 10.01 (mean ± SD), median 18.82, range 5.17–38.02 and a cut-off value for positivity SDs = 3.0. Sera from type 1 diabetic patients differed significantly from healthy human samples (unpaired t test with Welch correction, p < 0.0001). All patient sera became virtually negative (comparable to healthy individuals) under antigen excess: mean SDs changed from 19.26 to 1.29 (median 0.95, range − 1.20 to 8.75) with cold TrxZnT8 excess (Student t test for paired samples, p < 0.0001).

#### Immunochemical characterization of TrxZnT8-biotin

**Fig. 6:**
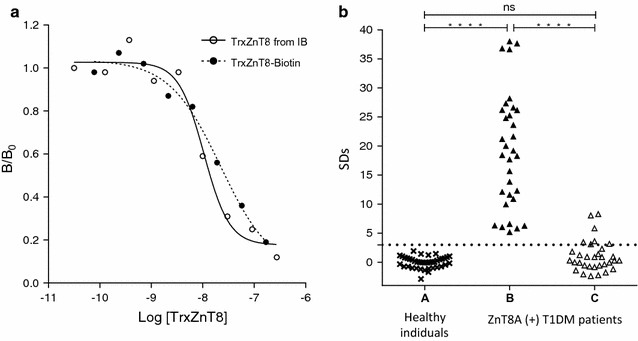
Immunochemical characterization of TrxZnT8-biotin. **a** Dose–response curves for a pool of 5 ZnT8A RBA positive type 1 diabetic patient sera for different concentrations of TrxZnT8 from IB (open circle, solid line) and TrxZnT8-biotin (closed circle, dotted line). **b** Inhibition capacity of TrxZnT8-biotin assessed in 32 ZnT8A RBA positive type 1 diabetic patient sera in the absence (B) or presence (C) of TrxZnT8-biotin. Fourty control sera were used in order to set a cut-off value (A). The binding was expressed as SDs, and the dotted line represents the cut-off value

Competitive quantitative and qualitative assays were also performed for TrxZnT8-biotin in order to evaluate any distortion in epitopes caused by biotynilation. The immunochemical identities between TrxZnT8 and TrxZnT8-biotin were analyzed and expressed in terms of parallelism. As seen in Fig. 6a, when using a pool of ZnT8A+ human sera, parallelism and identity between curves were achieved (one curve adequately fits all data, alpha = 0.05, R^2^ = 0.9673). The inhibition assay for TrxZnT8-biotin was performed by incubating 32 Type 1 diabetic patient sera in the presence of 280 nM TrxZnT8-biotin (Fig. 6b). All tested sera were ZnT8A positive with an SDs of 19.26 ± 10.01 (mean ± SD), median 18.82, range 11.07–38.02 and a cut-off value for positivity SDs = 3.0. It can be observed that sera from Type 1 diabetic patients differed significantly from healthy control samples (unpaired t test with Welch correction, p < 0.0001). All patient sera became virtually negative (comparable to healthy control individuals) under antigen excess: mean SDs changed from 19.21 to 1.03 (median 0.33, range − 2.38 to 8.30) with cold TrxZnT8-biotin excess (Student t test for paired samples, p < 0.0001).

### Immunoassay for ZnT8A detection using TrxZnT8

The preincubated-bridge ELISA (pb-ELISA) protocol was evaluated using human sera spanning a wide range of ZnT8A reactivity. In the pb-ELISA protocol the autoantibodies in serum samples (n = 62) bind divalently and form a bridge between immobilized TrxZnT8 and liquid phase biotinylated TrxZnT8.

The sensitivity was calculated as the percentage of type 1 diabetic patients that scored positive, and analytical sensitivity was calculated as the percentage of patients RBA positive that were positive by pb-ELISA. The specificity was calculated as 100% minus the percentage of normal human sera detected as positive.

**Fig. 7:**
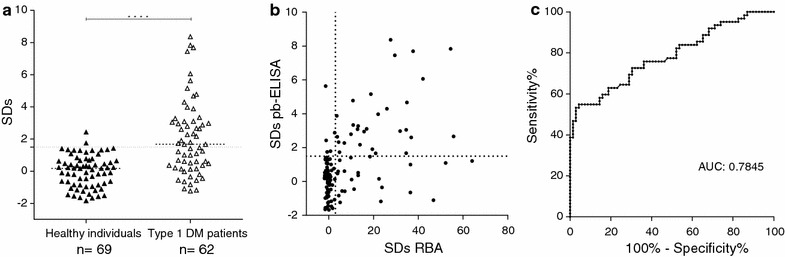
Immunoassays for ZnT8A detection in healthy human individuals (n = 69) and type 1 DM patients (n = 62). Results were expressed as SDs. **a** The results were obtained by pb-ELISA in both population. Medians for each population are indicated by a dashed line. **b** Correlation analysis of ZnT8A detection by RBA vs. pb-ELISA. In **a** and **b**, the dotted line represents the cut-off value for each assay. **c** ROC curve analysis of pb-ELISA; AUC was included

Results obtained by pb-ELISA are shown in Fig. 7a, signals were expressed as SDs. Out of 62 type 1 DM patients analyzed, 32 sera were positive (51.6% sensitivity), with SDs ranging from − 1.24 to 8.37, median 1.67 and cut-off value for positivity SDs = 1.5. Out of the 51 ZnT8A positive sera by RBA, 29 scored positive by pb-ELISA, whereas 22 were negatives. These results indicated that pb-ELISA had an analytical sensitivity of 56.9% for this sera collection. It is noteworthy that pb-ELISA detected positive 3 (4.8%) type 1 diabetic patients that RBA no. When evaluating healthy human sera, the specificity was 97.1%. Populations significantly differed for median antibody levels (Mann–Whitney *U* test, p < 0.0001).

Figure 7b depicts a statistical comparison of RBA and pb-ELISA results from diabetic patients and healthy individuals. Despite the differences in physical–chemical principles of the techniques, the correlation between both methods was significant (Spearman’s coefficient, rs = 0.399; p ˂ 0.001). Moreover, the agreement between the two immunoassays was 79.4%, with kappa statistic of 0.54 (Additional file 2: Table S1), representing good to fair agreement. Performance of pb-ELISA was analyzed using receiver operating characteristic (ROC) analysis and the area under the curve (AUC) of ROC curve was 0.7845 (Fig. 7c).

## Discussion

In previous works, it was described that the detection of ZnT8A, together with GADA, IA-2A and IAA, increases the diagnostic sensitivity of autoimmune DM [2, 15]. Unlike GAD and IA-2, ZnT8 is a highly specific β cell antigen; therefore, the presence of ZnT8A reveals, although indirectly, a specific damage of these cells. Even though radiometric methods have an excellent performance in the diagnostic support of DM, the employment of radioactive isotopes is falling out of use because of their multiple disadvantages (high costs, waste management difficulties, low applicability in laboratories, centralization of determinations, etc.). That is why, the search for inexpensive and reliable alternative assays for the detection of ZnT8A has been of crucial in the last few years. The methods most widely used include solid phase immunoassays which require large amounts of antigen for their development and routine application. In this sense, most extensively applied and widely used prokaryotic host for the production of recombinant proteins is the *E. coli* by diverse research groups and also in the private sectors [16, 17]. This expression system is simple, versatile due to the high amount of cloning vectors and host strains available, and implies a rapid generation of biomass at low cost.

Consistent with prior observations [5, 18], the ZnT8A workshop results highlighted the superior sensitivity of ZnT8-Arg-Trp325 assays measuring antibodies simultaneously to both arginine and tryptophan polymorphic variants compared to assays using each monomeric variant alone, a performance that was achieved without sacrificing specificity [13]. In addition, in a previous work we concluded that the heterodimeric construction ZnT8-Arg-Trp325 showed higher prevalence and signal levels and increased dynamic range in RBA for ZnT8A detection [2]. For these reasons, we decided to express this antigenic construction in *E. coli.* Cheng et al. expressed two C-terminal fragment of the ZnT8 antigen in *E. coli* alternatively containing Arg or Trp at position 325 [19]. Moreover, Kawasaki et al., expressed a dimeric C-terminal domain consisting of amino acids 275–369 [325Trp] and amino acids 275–369 [325Arg] with C-terminal 6-His tag in *Saccharomyces cerevisiae* strain c13ABYS86 using galactose for induction [20].

In this work, the heterodimeric construction of ZnT8 was successfully expressed as a fusion protein with thioredoxin in the ISF of *E. coli* strain GI698 at 20 °C. The thioredoxin moiety allowed the one-step purification by an arsine derivative affinity chromatography yielding ≈ 1.25 mg of protein/L of culture medium with 72.0–74.0% of purity. In addition, Trx-ZnT8 was recovered from IB of *E. coli* strain GI724 induced at 37 °C, subjected to in vitro refolding and purified by affinity chromatography, yielding ≈ 2.20 mg of protein/L of culture medium, 95.0–97.0% pure. Because the highest concentration and highest purity of the chimera was obtained from IB, these were selected as a more viable option for production of the recombinant protein. The amount of protein obtained with this methodology was suitable for the development of solid phase ZnT8A immunoassays; in fact, the result of a standard purification protocol may allow passive adsorption of more than 170 96-well polystyrene microplates. The first quality control step was to verify if TrxZnT8 had the theoretical expected MW. The analysis of the purified protein by SDS-PAGE and MS rendered results that coincided, within the experimental error, with the theoretical MW values calculated from the sequence. In addition, upon performing MS analysis after proteolytic digestion, sequence coverage of 73.6% was achieved over the 341 residues constituting the heterodymeric construction of TrxZnT8, which is a suitable percentage to accomplish the autoantigen identification.

Immunochemical studies of TrxZnT8 from ISF allowed us to demonstrate that the chimera was able to displace the binding of radiolabeled eukaryotic ZnT8 from ZnT8A, thus confirming the integrity of epitopes in the recombinant antigen, which is a critical requirement for ZnT8A recognition. It is noteworthy that the IC_50_ values obtained for 5 sera were in the range from 9.55 × 10^−9^ to 3.43 × 10^−8^ M, which correlates with literature data (**~** 1 × 10^−9^ M) [21]. Since the calculated IC_50_ for ZnT8A were, within the limits of experimental error, the same for TrxZnT8 and these previously published data for other variants of ZnT8 from *E. coli*, it can be speculated that the epitopes are not affected during prokaryotic expression and Trx fusion. Furthermore, with a larger number of ZnT8A positive patient sera, we demonstrated that they were able to recognize recombinant TrxZnT8, displacing the radioactively labeled eukaryotic antigen under TrxZnT8 excess. When TrxZnT8 was recovered from IB, immunochemical identity with the chimera obtained from ISF was demonstrated by RIA, according to the general principles of immunochemical cross reactivity [22]. Moreover, the calculated IC_50_ for 6 ZnT8A positive sera were similar to those obtained with TrxZnT8 from ISF. In addition, competitive qualitative assays showed that all patient sera became virtually negative under TrxZnT8 excess recovered from IB.

Since TrxZnT8 could be expressed in a simple prokaryotic system with high yields and purity, and it was properly identified and immunoreactive against ZnT8A positive patient sera, we proceeded to apply this protein in non-radiometric methods for ZnT8A detection. In order to increase the possibilities for immunoassay development, TrxZnT8 was biotinylated. When the immunochemical behavior of TrxZnT8-biotin was studied, the dose–response curves generated by RIA showed identity between TrxZnT8 and its biotinylated counterpart. Furthermore, competitive qualitative assays showed that almost all patient sera became negative under TrxZnT8-biotin excess. All these results indicate that the recognition of TrxZnT8 and the biotinylated protein by ZnT8A was the same; therefore, it could be deduced that the biotinylation did not alter critical epitopes for the interaction with ZnT8A.

Most conventional ELISA for measuring antibodies are based on surface-bound antigen and detection of total bound IgG with labeled xenogeneic anti-immunoglobulin antibodies. Frequently, the use of such labels increases the background- to- signal ratio. It was proposed that one way to reduce the background signal is to use the labeled specific antigen to detect bound antibody [23]. This also allows to employ undiluted sera increasing assay sensitivity. In the present work we proceeded to optimize pb-ELISA, the first described solid phase immunoassay with colorimetric detection for the determination of ZnT8A using a heterodimeric construction of C-terminal domain of ZnT8 (carrying Arg and Trp at position 325) expressed in *E. coli.* In this sense, it is important to emphasize that none of the commercial kits specify the antigenic construction employed. So it is not possible to establish if these kits detect antibodies directed against structural epitopes generated by ZnT8 dimerization, as it was previously demonstrated by our group [2].

The pb-ELISA protocol, based on ZnT8A crosslinking of immobilized TrxZnT8 and liquid-phase TrxZnT8-biotin, was highly specific (97.1%) but had lower sensitivity (51.6%) than RBA. The analytical sensitivity (56.9%) was calculated in order to compare the performance of pb-ELISA vs. the RBA, being the latter validated in the IASP 2012 and 2015 workshops. It is difficult to compare the performance of this assay with those of previous studies where ZnT8A is detected [19, 20] because different antigens were used, as well as different patient populations and healthy control individuals (with variables such as number, ethnicity and, possibly, sample matrix). The lack of correlation between solid-phase and liquid-phase assays has been previously reported and discussed [24–27]. In this sense, different thermodynamic principles apply to ELISA and RBA, since in the latter antigen–antibody reaction occurs in the liquid phase, in high dilution and near the equilibrium; meanwhile in ELISA the interaction occurs in solid phase, where the amount of immunocomplexes is highly dependent on antibodies concentration. In fact, in T1DM, autoantibodies concentration is really low (in the order of 10^−12^ M), turning their detection into an analytical challenge using solid phase assays, especially for sensitivity limitations. Then, as expected, there was a low correlation between the results obtained by ELISA protocol and RBA. However, despite the differences in the physical–chemical principles of the techniques, the agreement between the two immunoassays was 79.4%, with kappa statistic of 0.54. Moreover, the value of AUC achieved for pb-ELISA (0.7845) was considered acceptable.

Based on not requiring the use of radioisotopes and the semiautomated high-throughput assay format, the pb-ELISA should facilitate large-scale autoantibody screening. However, a weakness of the study is the limited number of subjects. A larger population should be ascertained in order to better characterize the performance of the immunoassay. In addition, it would be interesting to evaluate this assay in other patient populations such as adult-onset diabetic patients in order to better characterize the performance of the assay and its application in this group of patients.

These results may provide a basis for further research aimed at the simultaneous detection of multiple autoantibodies for the prediction, classification, monitoring, and prognosis of T1DM. In this sense, methods for ZnT8A detection by flow cytometry based on the use of polystyrene microspheres are currently being developed in our laboratory [28]. This assay has the potential of simultaneous determination of the main humoral markers in autoimmune DM. Therefore, multiplex testing will facilitate high-throughput screening of T1DM in the general population.

## Conclusions

The approach reported herein should be accessible to many laboratories and allow the production of TrxZnT8 at low cost and effort. In turn, the availability of properly folded ZnT8 would encourage researchers to improve present and develop new, low-cost tests for ZnT8A detection as well as in clinical trials with ZnT8 immunomodulation.

## Methods

### Sera collection

#### Human sera collection

Sera were obtained from 83 selected type 1 diabetic patients spanning a wide range of ZnT8A reactivity. This group included Argentinian children and adolescents admitted to the Nutrition Service at Gutierrez National Pediatric Hospital (Buenos Aires, Argentina) from May 2013 to April 2016, with a mean age of 9.8 years at diagnosis, median age of 9.0, range 2–17 years and male/female: 44/39. Serum samples were collected before or within 72 h of starting insulin treatment. Type 1 DM was diagnosed according to WHO criteria [29]. The protocol was approved by the Ethical Committee of the Gutierrez National Pediatric Hospital and parental consent were obtained.

Healthy human sera (*n* = 70, mean age of 29.7 years with median age of 25, range 16–80 and male/female: 36/34) were obtained from Argentinian healthy subjects without personal or family history of DM or autoimmune disease. The collection of serum samples was approved by the Ethics Committee of the José de San Martín Clinical Hospital, University of Buenos Aires (UBA), Buenos Aires, Argentina. All subjects were informed about the purpose of the study, and a signed consent for study participation was obtained.

#### Rabbit polyclonal sera against Trx

Antibodies to Trx were obtained by immunizing two New Zealand white rabbits with 1 mg of recombinant Trx emulsified in complete Freund’s adjuvant. The initial injection was followed by booster injections with 1 mg of the mentioned protein in incomplete Freund’s adjuvant at 3-week intervals. The rabbits were bled 15 days after booster dose. The immunoreactivity of polyclonal sera to Trx was tested by indirect-ELISA using Trx-coated polystyrene plates and by western blot. All animals were housed under specific conditions according to the “Guide for the Care and Use of Laboratory Animals” by the National Research Council of the National Academies (USA); experiments were performed in compliance with the Argentinian animal protection laws and approved by the “Prof. Ricardo A. Margni” Humoral Immunity Studies Institute (IDEHU), National Research Council (CONICET-UBA).

### Expression of TrxZnT8 in *Escherichia coli*

#### Expression vector

Unless otherwise indicated, standard molecular biology protocols were used according to Sambrook et al. [30]. Restriction enzymes, *Pfu* polymerase and *T4* ligase were from Promega (Madison, WI, USA). The coding sequence of dimeric C-terminal domain of human ZnT8 consisting of amino acids 268–369 [Arg325] and amino acids 268–369 [Trp325] were optimized for prokaryotic expression. The ZnT8 optimized nucleotide sequence was synthesized by GenScript (GenScript Corporation, Piscataway, NJ, USA; http://www.GenScript.com) including *Kpn*I and *Xba*I sites at the 3′ and 5′ ends, respectively. The synthesized construct (682 bp) was obtained from GenScript in plasmid pUC57 and maintained in *E. coli* strain JM109 (Promega, Madison, WI, USA). After propagation and purification of the vector with QIAprep spin Miniprep Kit (QIAGEN, Hilden, Germany), ZnT8 construct was digested with *Kpn*I and *Xba*I and ligated into the pTrxFus linearized vector (Invitrogen, Carlsbad, CA, USA). The quality of the new vector encoding the fusion protein TrxZnT8 (Additional file 3: Figure S2) was corroborated by sequencing (Macrogen Inc, Seoul, Korea).

### *Escherichia coli* transformation and protein expression

Competent *E. coli* strains GI724 (ATCC^®^ 55151™) and GI698 (Invitrogen, Carlsbad, CA, USA) were transformed by electroporation with pTrxZnT8. Bacteria were cultured at 30 °C in 0.2% casein amino acids, 0.5% glucose, 1 mM MgCl_2_ and 100 µg/mL ampicillin, and protein expression was induced with 100 µg/mL Trp at 37 °C for GI724 or 20 °C for GI698, at different times (1.5, 3.0 and 16.0 h).

### *Escherichia coli* disruption and intracellular protein isolation

*Total cell lysate* (TCL) Bacteria from 1 mL culture were collected by centrifugation, suspended in 0.2 mL of sodium dodecyl sulphate-polyacrylamide gel electrophoresis (SDS-PAGE) sample buffer (0.05 M Tris–HCl, 12.0% glycerol, 0.005% bromophenol blue, 4.0% SDS, 4.0% 2-mercaptoethanol—2ME, pH 6.8) and boiled for 5 min.

*Intracellular soluble fraction* (ISF) Bacteria from 200 mL culture were collected by centrifugation, suspended in 4 mL of lysis buffer (50 mM Tris–HCl, 100 mM NaCl, 1 mM EDTA, pH 7.0) and sonicated in the presence of 1 mM 2ME and protease inhibitors (0.1% aprotinin and 2 mM phenylmethylsulphonyl fluoride). After sonication, Triton X-100 was added to a final concentration of 0.1%, and the mixture was incubated for 10 min at 0 °C. The ISF was separated from the IB by centrifugation at 15,000×*g* for 10 min at 4 °C.

*Inclusion bodies* (IB) IB from 200 mL of culture were washed with 2 M urea in 0.1 M Tris, pH 8.5, and solubilized with 8 M urea in 0.1 M Tris, pH 8.5. Oxidative refolding was initiated by dialysis at 4 °C against 0.5 M l-arginine, 50 mM Tris–HCl, 5 mM EDTA, 5 mM reduced glutathione and 0.5 mM oxidized glutathione, pH 9.5 [31, 32].

### Purification of TrxZnT8 by affinity chromatography

#### Purification of TrxZnT8 from the Intracellular soluble fraction

TrxZnT8 from the ISF was purified by means of affinity chromatography following the protocol previously described [31, 33]. Briefly, the resin was based on an agarosa support covalently modified with phenylarsine oxide, which permitted the binding of proteins containing vicinal dithiol residues, as it occurs in Trx [34]. The lysate (4 mL) was added to the resin, previously equilibrated in lysis buffer (≈ 4 mL) and activated with 4 column volumes (CV) of lysis buffer containing 20 mM 2ME, and the resulting suspension was incubated for 1.5 h at 4 °C. The resin was poured into a column and washed sequentially with 6 CV of lysis buffer, 6 CV of lysis buffer containing 1 mM 2ME and 3 CV of lysis buffer containing 5 mM 2ME. Bound proteins were eluted with several 2 mL aliquots of lysis buffer containing 100 mM 2ME. The protein concentration in purified fractions was determined using the Coomasie Plus (Bradford) Assay kit. Aprotinin was added to a final concentration of 0.1%.

#### Purification of TrxZnT8 from the inclusion bodies

After overnight (ON) refolding of IB, the buffer was exchanged by gel filtration on a PD10 column (Amersham Biosciences, Piscataway, NJ) equilibrated with phosphate saline buffer (PBS, 1.5 mM KH_2_PO_4_, 8.1 mM Na_2_HPO_4_, 0.14 M NaCl, 2.7 mM KCl, pH 7.4) and TrxZnT8 was purified by affinity chromatography. Briefly, the resin was based on Anti-Trx Ig-Sepharose CL-4B prepared by coupling purified Anti-Trx immunoglobulins to Sepharose CL-4B activated with CNBr (Pharmacia-LKB Biotechnology, Uppsala). TrxZnT8 from IB (2 mL) was added to the PBS equilibrated resin (2 mL) and the suspension was incubated ON at 4 °C. The resin was poured into a column and washed with 10 CV of PBS. Elution was performed with 0.53% diethylamine in distilled water, pH 11.0; 2 mL fractions were collected in tubes containing 2 mL of 1 M Tris–HCl pH 7.4 to neutralize the eluates and buffer exchange to PBS was made by using ZEBA desalt spin column (Pierce Biotechnology, Rockford, IL, USA) according to the manufacturer’s instructions.

The protein concentration in purified fractions was determined using the Coomasie Plus (Bradford) Assay kit. Aprotinin was added to a final concentration of 0.1%.

### Biotinylation of TrxZnT8

Two mL of the purified fusion protein from IB were subjected to buffer exchange to PBS using a ZEBA desalt spin column (Pierce Biotechnology, Rockford, IL, USA) according to the manufacturer’s instructions. The desalted protein was then incubated for 2 h at 0 °C with a 300-fold molar excess of sulfo-NHS-biotin (Pierce Biotechnology, Rockford, IL, USA). Free biotin was removed on a new ZEBA desalt spin column.

### Biochemical characterization of TrxZnT8

#### Molecular weight determination by SDS-PAGE

The general SDS-PAGE procedure for MW estimation consisted in separating several conventional standard proteins with known MW (Amersham Pharmacia Biotech, Little Chalfont Buckinghamshire, England) in parallel with a TrxZnT8 sample in a 10.0% T, 6.0% C acrylamide gel with 1.5 mm thickness and under reducing conditions [35]. The standard proteins were used in order to generate a curve correlating MW and migration in the gel (relative mobility or R_f_), from which the MW of TrxZnT8 sample was estimated.

#### Mass spectrometry analysis

In order to assess total protein MW, affinity purified TrxZnT8 from ISF was subjected to mass spectrometry (MS) analysis. One µL aliquot of the sample of TrxZnT8 was spotted onto an AnchorChip (Bruker, Billerica, MA, USA). One µL of an oversaturated solution of sinapinic acid in 30/70/0.1% acetonitrile/water/TFA was added to the sample and left to crystallize by air-drying. Samples were analyzed on a Bruker Microflex MALDI-TOF (Bruker, Billerica, MA, USA). Aiming to further characterize and identify the protein, TrxZnT8 was subjected to proteolytic digestion and MS analysis. Concisely, the sample was dissolved in 50 mM NH_4_HCO_3_ buffer, pH 8.0; a volume equivalent to 20 µg was subjected to disulfide bond reduction, with 20 mM dithiothreitol for 45 min at 56 °C, and alkylation with 20 mM iodoacetamide for 45 min at room temperature (RT) in the dark. The sample was then diluted to a final concentration of 1 M urea. Finally, trypsin and chymotrypsin proteolytic digestion were performed separately and samples were analyzed by nanoHPLC (EASY-Spray Accucore, Thermo scientific, West Palm Beach, FL, USA) coupled to a mass spectrometer with Orbitrap technology (Q-Exactive, Thermo Scientific, West Palm Beach, FL, USA; at Centro de Estudios Químicos y Biológicos por Espectrometría de Masa-CEQUIBIEM- CONICET-UBA, Argentina), enabling both separation and identification of peptides. Ionization of samples was made by electrospray (EASY-SPRAY, Thermo Scientific, West Palm Beach, FL, USA) and data analysis was performed by the Proteome Discoverer software version 1.4, Thermo Scientific. Coverage percentages were calculated based on the number of identified peptides/total number of peptides.

### Immunochemical characterization of TrxZnT8

#### Western blot analysis

Total *E. coli* lysates, ISF and IB were analyzed by SDS-PAGE [35] and western blot (WB). For comparison, all SDS-PAGE lanes in each gel contained proteins recovered from the same amount of cells. Protein bands were transferred to nitrocellulose membranes and unoccupied binding sites were blocked by incubation with 2.0% skim milk in Tris buffer saline (TBS, 0.05 M Tris–HCl, 0.15 M NaCl, pH 7.5) for 2 h at RT. After 3 washing steps with 0.05% Tween 20 in TBS (TBS-T), membranes were incubated ON at RT with polyclonal sera to Trx diluted 1/200 in 2.0% skim milk, 0.05% Tween 20 in TBS (TBS-MT) and then washed five times with TBS-T. Bound antibodies were visualized by incubation with horseradish peroxidase (HRP)-conjugated goat antibodies to rabbit IgG (Jackson ImmunoResearch Laboratories, Inc., West Grove, PA) diluted 1/2000 in TBS-MT, followed by the addition of alpha-chloronaphthol (Sigma-Aldrich, Inc., St Louis, MO) and 10 vol H_2_O_2_.

#### Qualitative and quantitative competition assays

##### Radioimmunoassay protocol

Quantitative competition assays were performed by standard Radioimmunoassays (RIA). The [^35^S]ZnT8 tracer was obtained by in vitro transcription/translation of the _c_DNA for the dimeric construct encoding C-terminal domain of human ZnT8, with amino acids 268–369 [Arg325] and amino acids 268–369 [Trp325], using a rabbit reticulocyte lysate system (Promega, Madison, WI, USA) in the presence of [^35^S]-methionine (New England, Nuclear, Boston, MA, USA), according to the manufacturer´s instructions [2, 12]. The RIA was carried out by incubating 5 µL of ZnT8A(+) type 1 diabetic patient sera in duplicate with 10,000 cpm of [^35^S]ZnT8 in the presence of serial concentrations (37.0 pM–2.2 µM) of purified TrxZnT8 from ISF or IB in a final volume of 60 µL. After ON incubation, 50 μL of 40.0% protein A-Sepharose 4B FF (GE Healthcare Biosciences, Uppsala, Sweden) in RIA buffer (0.02 M Tris–HCl, 0.15 M NaCl, 0.1% Tween 20, pH 7.4) were added and incubated for 2 h at RT on an end-over-end shaker. Subsequently, samples were allowed to settle and the supernatants were discarded in order to isolate immunocomplexes. Pellets were washed three times with 200 µL of RIA buffer and once with 200 µL of 0.2 M NaCl in RIA buffer. Finally, pellets were suspended in 100 µL of 1.0% SDS and supernatants were carefully transferred to vials for scintillation counting (1 min/tube). Results for each sample were calculated as Bound % (B %) = 100 × (bound cpm/total cpm). Inhibitory Dose–response curves [log (inhibitor) vs. response–variable slope (four parameters)] were fitted to the mathematical function:

$${\text{B}}/{\text{B}}_{0} = {\text{ B}}/{\text{B}}_{0\text{min} } + \, ({\text{B}}/{\text{B}}_{0\text{max} } - {\text{ B}}/{\text{B}}_{0\text{min} } )/ \left( { 1+ 10^{{^{{\left[ {({\text{log IC5}}0 - {\text{log TrxZnT8 dose}})*{\text{Hill Slope}}} \right]}} }} } \right)$$

where B corresponds to B% measurements, B_0_ is the B% at zero concentration of unlabelled antigen, B/B_0min_ and B/B_0max_ are the minimal and maximal response, respectively and the parameter IC_50_ represents the concentration of TrxZnT8 that gave a response half between B/B_0min_ and B/B_0max_. Hill Slope describes the steepness of the family of curves. The same protocol was performed for TrxZnT8-biotin, in serial concentrations of 172.0 pM–0.2 µM.

##### Inhibition assay

The ability of TrxZnT8 to compete with [^35^S]ZnT8 for the binding to antibodies was assessed qualitatively by incubating sera from 30 ZnT8A(+) type 1 diabetic patients with the tracer in the presence of purified fusion protein, either from the ISF (190 nM) or recover from IB (290 nM). Briefly, 5 µL of human sera were incubated ON at 4 °C with 10,000 cpm of [^35^S]ZnT8 in the absence or presence of unlabelled TrxZnT8 in a final volume of 60 µL in RIA buffer. Immunocomplexes were isolated with protein A-Sepharose 4B FF; pellets were washed and suspended in 1.0% SDS, as described in the RIA protocol. The radioactivity of supernatants was counted. Results for each sample (with or without TrxZnT8) were calculated as B %, and expressed as Standard Deviation scores (SDs) = (B% − B_c_%)/SD_c_, where B_c_% is the mean B% of healthy human sera and SD_c_ its standard deviation. The same protocol was followed for TrxZnT8-biotin in the presence of antigen excess (280 nM).

### ZnT8A detection by radioligand binding assay

ZnT8A were assessed by radioligand binding assay (RBA) as previously described [2, 12]. Briefly, 5 µL of human sera were incubated ON at 4 °C with 10,000 cpm of the [^35^S]ZnT8 in a final volume of 60 µL in RIA buffer. Subsequently, isolation of immune complexes was carried out by addition of 50 μL 40% protein A-Sepharose 4B FF in RIA buffer. Pellets were washed three times with 200 μL of RIA buffer and once with 200 μL of 0.2 M NaCl in RIA buffer. Finally, pellets were suspended in 100 μL of 1% SDS and supernatants were carefully transferred to vials for scintillation counting. Results were expressed as precision units, SDs. Thirty healthy human sera were included in each assay demonstrating that *B*_C_% was normally distributed. The whole procedure was validated in the Islet Autoantibody Standardization Program (IASP) 2015 where our laboratory achieved 68% sensitivity and 97.8% specificity (Lab. 0519).

### ZnT8A detection by preincubated bridge ELISA (pb-ELISA)

#### Reagents

The coating buffer was PBS, the blocking buffer was 2% skim milk in PBS, the washing buffer was PBS containing 0.05% Tween 20 (PBS-T) and the preincubation buffer was 0.1% aprotinin and 2% skim milk in PBS-T. Reagent dilutions were prepared in 2% skim milk, in PBS-T (PBS-MT). Avidin– HRP was purchased from Jackson ImmunoResearch Laboratories, Inc. The 3,3′,5,5′-tetramethyl-benzidine/H_2_O_2_ (Single Component TMB Peroxidase EIA Substrate Kit, BioRad, Hercules, CA, USA) was employed as the chromogenic substrate. Except when otherwise indicated, incubations were performed at RT, washing steps were performed with PBS-T and 50 μL per well were added in each incubation step.

#### pb-ELISA protocol

The protocol employed was based on those previously described [20, 36], with minor modifications. Briefly polystyrene microplates (Maxisorp, NUNC, Rorkilde, Denmark) were coated ON at 4 °C with 0.2 µg of purified TrxZnT8 from IB per well, washed three times with PBS, blocked for 1.5 h with 200 µL of blocking solution per well, and washed five times. Separately, in Eppendorf tubes a sample volume of 40 µL was incubated ON at 4 °C with 20 µL of preincubation buffer containing 3.8 ng of TrxZnT8-biotin. Then, the preincubates were transferred to the microplates coated with TrxZnT8 and incubated for 1.5 h. Microplates were washed five times, and bound TrxZnT8-biotin was detected by the addition of Avidin-HRP diluted 1/600. After washing (four times plus one final wash with PBS), the chromogenic substrate was added and plates were incubated in the dark. The colour reaction was stopped with 4 N H_2_SO_4._

### Statistical analysis

The statistical analysis was performed using GraphPad Prism software, version 6.01 for Windows (GraphPad Software, San Diego California, USA, http://www.graphpad.com). The immunochemical identities and parallelism between inhibitory dose–response curves obtained with TrxZnT8 from ISF and from IB and TrxZnT8-biotin were analyzed by comparing B/B_0min_, B/B_0max_, Hill slope and IC_50_ values by the extra sum-of-squares F test comparison method. Normal distribution of data was analyzed by the D’Agostino and Pearson omnibus normality test. The selection of optimal cut-off values was based on curves constructed by plotting the calculated specificity and sensitivity of each protocol against the cut-off values. Statistical significance was evaluated using parametric tests: paired samples Student *t* test and unpaired samples Student *t* test with Welch correction, or non-parametric tests: Wilcoxon matched-pairs signed rank test or Mann–Whitney *U* test for unpaired data, when applicable. Performance of pb-ELISA was analyzed using ROC analysis and the AUC was evaluated. Spearman coefficient of correlation (rs) was calculated to evaluate inter-assay correlation. The kappa statistic was used to measure the strength of agreement between the pb-ELISA and RBA results, with a kappa statistic value of 0.75 representing excellent agreement, 0.40–0.75 representing good to fair agreement and 0.40 representing poor agreement [37, 38].

## Additional files

**Additional file 1: Figure S1.** ZnT8 expression as a fusion protein with Trx in *E. coli* strain GI698 (a and c) and strain GI724 (b and d). (a) and (b): SDS-PAGE (12.1% T, 6.0% C, 1 mm, under reducing conditions, stained with Coomassie Brillant Blue R-250), (c) and (d): WB revealed with a rabbit polyclonal serum to thioredoxin as primary antibody. Lanes 1–4: samples from *E. coli* strain GI698 and GI724 transformed with pTrxZnT8. Lanes 5–8: samples from untransformed *E. coli* strain GI698 and GI724. Lanes 1 and 5: total cell lysates before induction (0 h); lanes 2 and 6: total cell lysates after 3.0 h of induction; lanes 3 and 7: intracellular soluble fractions after 3.0 of induction; lanes 4 and 8: Inclusion bodies after 3.0 of induction. Arrows indicate the electrophoretic mobility of TrxZnT8.

**Additional file 2: Table S1.** Agreement between the pb-ELISA and RBA with sera from type 1 diabetic patients (n = 62) and control individuals (n = 69). The agreement between the two immunoassays was 79.4%, with kappa statistic of 0.54.

**Additional file 3: Figure S2.** Map of the vector constructed for the expression of TrxZnT8 in *E. coli*. The ZnT8 sequence was inserted into the multiple cloning site of the expression vector and expressed as an amino terminal fusion to the *E. coli* protein thioredoxin. To drive expression of thioredoxin fusions, pTrxFus uses the pL promoter from the λ bacteriophage and the AspA transcription terminator. Plasmid selection and maintenance was ensured by the presence of a beta-lactamase gene (BLA) that provide ampicillin resistance. *Kpn*I and *Xba*I sites are indicated at the 3´ and 5´ ends of the ZnT8 sequence. RBS: ribosome binding site. EK site: enterokinase cleavage site.

## Abbreviations

2ME: 2-mercaptoethanol
Arg: arginine
AUC: area under curve
B%: bound%
CV: column volume
DASP: diabetes antibody standardization program
DM: diabetes mellitus
GADA: glutamic acid decarboxylase autoantibodies
HRP: horseradish peroxidase
IA-2A: insulinoma associated protein tyrosine phosphatase 2 autoantibodies
IAA/PAA: insulin/proinsulin autoantibodies
IB: inclusion bodies
IC50: concentration that caused 50% inhibition
ISF: intracellular soluble fraction
LADA: latent autoimmune diabetes of the adults
MS: mass spectrometry
MW: molecular weights
ON: overnight
pb-ELISA: preincubated bridge ELISA
PBS: phosphate saline buffer
PBS-T: 0.05% Tween 20 in PBS
RBA: radioligand binding assay
RIA: radioimmunoassay
ROC: receiver operating characteristic
RT: room temperature
SDs: standard deviation scores
SDS-PAGE: sodium dodecyl sulphate-polyacrylamide gel electrophoresis
SNP: single nucleotide polymorphism
TBS: tris buffer saline
TBS-MT: 2.0% skim milk in TBS-T
TBS-T: 0.05% Tween 20 in TBS
TCL: total cell lysate
Trp: tryptophan
Trx: thioredoxin
TrxZnT8: zinc transporter 8 fused to thioredoxin
WB: western blot
ZnT8: zinc transporter 8
ZnT8A: zinc transporter 8 autoantibodies

## References

[1] Wenzlau JM, Juhl K, Yu L, Moua O, Sarkar SA, Gottlieb P (2007). The cation efflux transporter ZnT8 (Slc30A8) is a major autoantigen in human type 1 diabetes. Proc Natl Acad Sci USA.

[2] Faccinetti NI, Guerra LL, Steinhardt AP, Iacono RF, Frechtel GD, Trifone L (2016). Characterization of zinc transporter 8 (ZnT8) antibodies in autoimmune diabetic patients from Argentinian population using monomeric, homodimeric, and heterodimeric ZnT8 antigen variants. Eur J Endocrinol.

[3] Chimienti F, Devergnas S, Favier A, Seve M (2004). Identification and cloning of a beta-cell-specific zinc transporter, ZnT-8, localized into insulin secretory granules. Diabetes.

[4] Sladek R, Rocheleau G, Rung J, Dina C, Shen L, Serre D (2007). A genome-wide association study identifies novel risk loci for type 2 diabetes. Nature.

[5] Kawasaki E, Uga M, Nakamura K, Kuriya G, Satoh T, Fujishima K (2008). Association between anti-ZnT8 autoantibody specificities and SLC30A8 Arg325Trp variant in Japanese patients with type 1 diabetes. Diabetologia.

[6] Kawasaki E, Nakamura K, Kuriya G, Satoh T, Kobayashi M, Kuwahara H (2011). Zinc transporter 8 autoantibodies in fulminant, acute-onset, and slow-onset patients with type 1 diabetes. Diabetes Metab Res Rev.

[7] Andersen MK, Harkonen T, Forsblom C, Groop PH, Knip M, Tuomi T (2013). Zinc transporter type 8 autoantibodies (ZnT8A): prevalence and phenotypic associations in latent autoimmune diabetes patients and patients with adult onset type 1 diabetes. Autoimmunity.

[8] Vaziri-Sani F, Oak S, Radtke J, Lernmark K, Lynch K, Agardh CD (2010). ZnT8 autoantibody titers in type 1 diabetes patients decline rapidly after clinical onset. Autoimmunity.

[9] De Grijse J, Asanghanwa M, Nouthe B, Albrecher N, Goubert P, Vermeulen I (2010). Predictive power of screening for antibodies against insulinoma-associated protein 2 beta (IA-2beta) and zinc transporter-8 to select first-degree relatives of type 1 diabetic patients with risk of rapid progression to clinical onset of the disease: implications for prevention trials. Diabetologia.

[10] Fabris M, Zago S, Liguori M, Trevisan MT, Zanatta M, Comici A (2015). Anti-zinc transporter protein 8 autoantibodies significantly improve the diagnostic approach to type 1 diabetes: an Italian multicentre study on paediatric patients. Auto Immun Highlights.

[11] Gomes KF, Semzezem C, Batista R, Fukui RT, Santos AS, Correia MR (2017). Importance of zinc transporter 8 autoantibody in the diagnosis of type 1 diabetes in Latin Americans. Sci Rep.

[12] Trabucchi A, Faccinetti NI, Guerra LL, Puchulu FM, Frechtel GD, Poskus E (2012). Detection and characterization of ZnT8 autoantibodies could help to screen latent autoimmune diabetes in adult-onset patients with type 2 phenotype. Autoimmunity.

[13] Lampasona V, Schlosser M, Mueller PW, Williams AJ, Wenzlau JM, Hutton JC (2011). Diabetes antibody standardization program: first proficiency evaluation of assays for autoantibodies to zinc transporter 8. Clin Chem.

[14] Goetz H, Kuschel M, Wulff T, Sauber C, Miller C, Fisher S (2004). Comparison of selected analytical techniques for protein sizing, quantitation and molecular weight determination. J Biochem Biophys Methods.

[15] Yi B, Huang G, Zhou ZG (2015). Current and future clinical applications of zinc transporter-8 in type 1 diabetes mellitus. Chin Med J.

[16] Baneyx F (1999). Recombinant protein expression in *Escherichia coli*. Curr Opin Biotechnol.

[17] Pines O, Inouye M (1999). Expression and secretion of proteins in *E. coli*. Mol Biotechnol.

[18] Wenzlau JM, Walter M, Gardner TJ, Frisch LM, Yu L, Eisenbarth GS (2010). Kinetics of the post-onset decline in zinc transporter 8 autoantibodies in type 1 diabetic human subjects. J Clin Endocrinol Metab.

[19] Cheng L, Li T, Zhang D, Chen B (2015). Prokaryotic expression of bioactive zinc transporter 8 antigens and detection of diabetes specific autoantibodies in a single dot immunogold filtration assay. Clin Lab.

[20] Kawasaki E, Tanaka M, Miwa M, Abiru N, Kawakami A (2014). Novel enzyme-linked immunosorbent assay for bivalent ZnT8 autoantibodies. Acta Diabetol.

[21] Skarstrand H, Krupinska E, Haataja TJ, Vaziri-Sani F, Lagerstedt JO, Lernmark A (2015). Zinc transporter 8 (ZnT8) autoantibody epitope specificity and affinity examined with recombinant ZnT8 variant proteins in specific ZnT8R and ZnT8W autoantibody-positive type 1 diabetes patients. Clin Exp Immunol.

[22] Berzofsky JA, Schechter AN (1981). The concepts of crossreactivity and specificity in immunology. Mol Immunol.

[23] Brooking H, Ananieva-Jordanova R, Arnold C, Amoroso M, Powell M, Betterle C (2003). A sensitive non-isotopic assay for GAD65 autoantibodies. Clin Chim Acta.

[24] Sodoyez-Goffaux F, Koch M, Dozio N, Brandenburg D, Sodoyez JC (1988). Advantages and pitfalls of radioimmune and enzyme linked immunosorbent assays of insulin antibodies. Diabetologia.

[25] Palmer JP, Wilkin TJ, Kurtz AB, Bonifacio E (1990). The third international workshop on the standardisation of insulin autoantibody measurement. Diabetologia.

[26] Levy-Marchal C, Bridel MP, Sodoyez-Goffaux F, Koch M, Tichet J, Czernichow P (1991). Superiority of radiobinding assay over ELISA for detection of IAAs in newly diagnosed type I diabetic children. Diabetes Care.

[27] Berzofsky JA, Berkower IJ, Epstein SL, Paul WE (1993). Antigen–antibody interactions and monoclonal antibodies. Fundamental immunology.

[28] Guerra LL, Trabucchi A, Faccinetti NI, Iacono RF, Ureta DB, Poskus E (2014). Flow cytometric microsphere-based immunoassay as a novel non-radiometric method for the detection of glutamic acid decarboxylase autoantibodies in type 1 diabetes mellitus. Analyst.

[29] World Health Organization. Diabetes mellitus. Report of a WHO Study Group. World Health Organization Technical Report Service 1985, vol. 727. p. 1–113. 3934850

[30] Sambrook J, Fritsch EF, Maniatis T (1989). Molecular cloning: a laboratory manual.

[31] Trabucchi A, Guerra LL, Faccinetti NI, Iacono RF, Poskus E, Valdez SN (2012). Expression and characterization of human proinsulin fused to thioredoxin in *Escherichia coli*. Appl Microbiol Biotechnol.

[32] Valdez SN, Iacono RF, Villalba A, Landaburu AC, Ermacora MR, Poskus E (2003). A radioligand-binding assay for detecting antibodies specific for proinsulin and insulin using 35S-proinsulin. J Immunol Methods.

[33] Guerra LL, Faccinetti NI, Trabucchi A, Rovitto BD, Sabljic AV, Poskus E (2016). Novel prokaryotic expression of thioredoxin-fused insulinoma associated protein tyrosine phosphatase 2 (IA-2), its characterization and immunodiagnostic application. BMC Biotechnol.

[34] Hoffman RD, Lane MD (1992). Iodophenylarsine oxide and arsenical affinity chromatography: new probes for dithiol proteins. Application to tubulins and to components of the insulin receptor-glucose transporter signal transduction pathway. J Biol Chem.

[35] Shägger H, von Jagow G (1987). Tricine-sodium dodecyl sulfate-polyacrylamide gel electrophoresis for the separation of proteins in the range from 1 to 100 kDa. Anal Biochemi.

[36] Villalba A, Valdez SN, Iacono RF, Poskus E (2007). Development of 2 alternative enzyme-linked immunosorbent assays for routine screening of glutamic acid decarboxylase autoantibodies. Clin Chim Acta.

[37] Cohen J (1968). Weighted kappa: nominal scale agreement with provision for scaled disagreement or partial credit. Psychol Bull.

[38] Cohen J (1960). A coefficient of agreement for nominal scales. Educ Psychol Meas.

