# Supplementary figures and images for: Prokaryotic expression and characterization of the heterodimeric construction of ZnT8 and its application for autoantibodies detection in diabetes mellitus
Source: Microb Cell Fact. 2017 Nov 13;16:196. doi: 10.1186/s12934-017-0816-4 (PMC5683521; doi:10.1186/s12934-017-0816-4)

## Slide 1
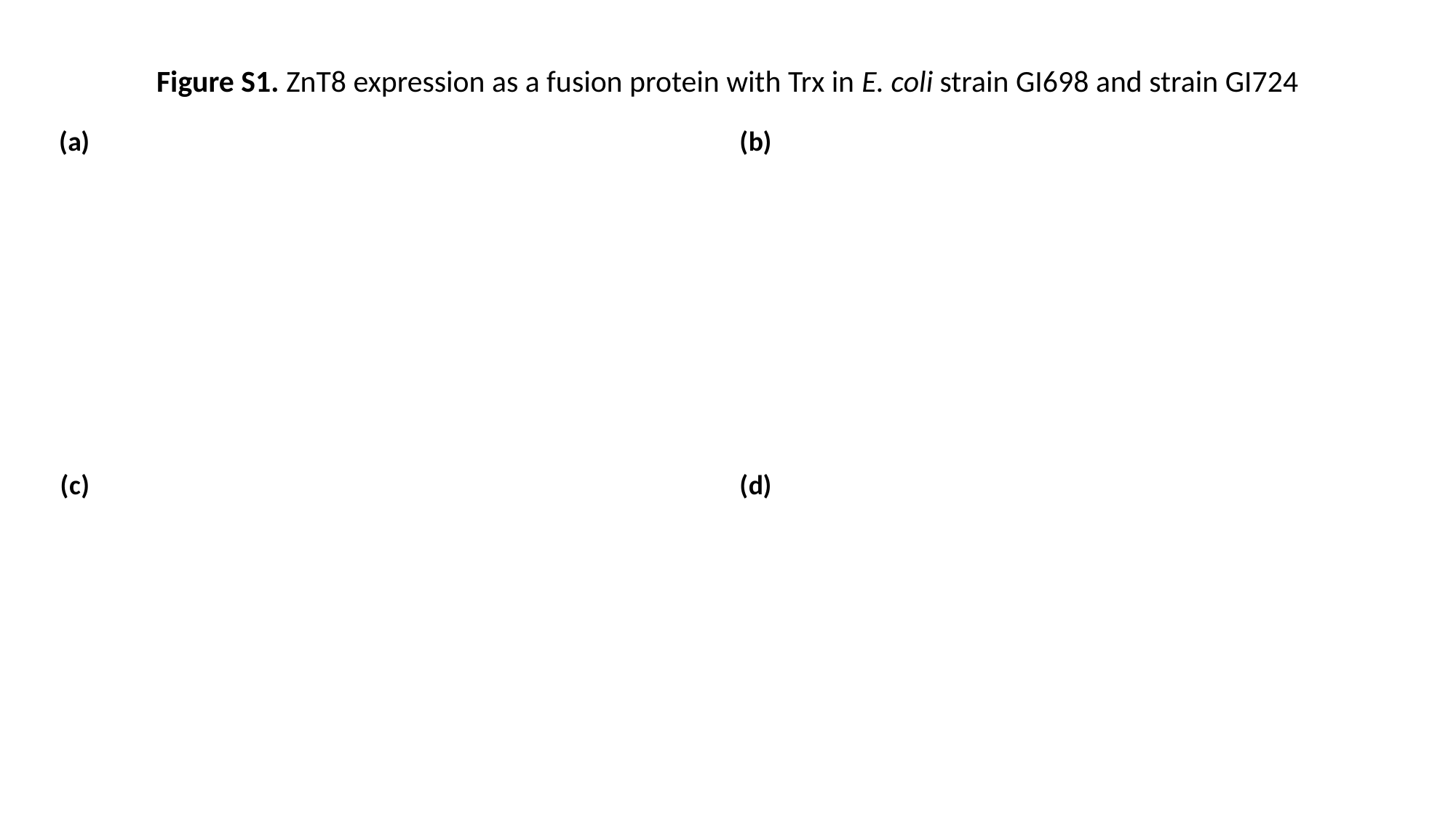

Figure S1. ZnT8 expression as a fusion protein with Trx in E. coli strain GI698 and strain GI724

Supplement: Supplementary file 1 — Additional file 1: Figure S1. ZnT8 expression as a fusion protein with Trx in E. coli strain GI698 (a and c) and strain GI724 (b and d). (a) and (b): SDS-PAGE (12.1% T, 6.0% C, 1 mm, under reducing conditions, stained with Coomassie Brillant Blue R-250), (c) and (d): WB revealed with a rabbit polyclonal serum to thioredoxin as primary antibody. Lanes 1–4: samples from E. coli strain GI698 and GI724 transformed with pTrxZnT8. Lanes 5–8: samples from untransformed E. coli strain GI698 and GI724. Lanes 1 and 5: total cell lysates before induction (0 h); lanes 2 and 6: total cell lysates after 3.0 h of induction; lanes 3 and 7: intracellular soluble fractions after 3.0 of induction; lanes 4 and 8: Inclusion bodies after 3.0 of induction. Arrows indicate the electrophoretic mobility of TrxZnT8. [file 12934_2017_816_MOESM1_ESM.pptx]

## Slide 1
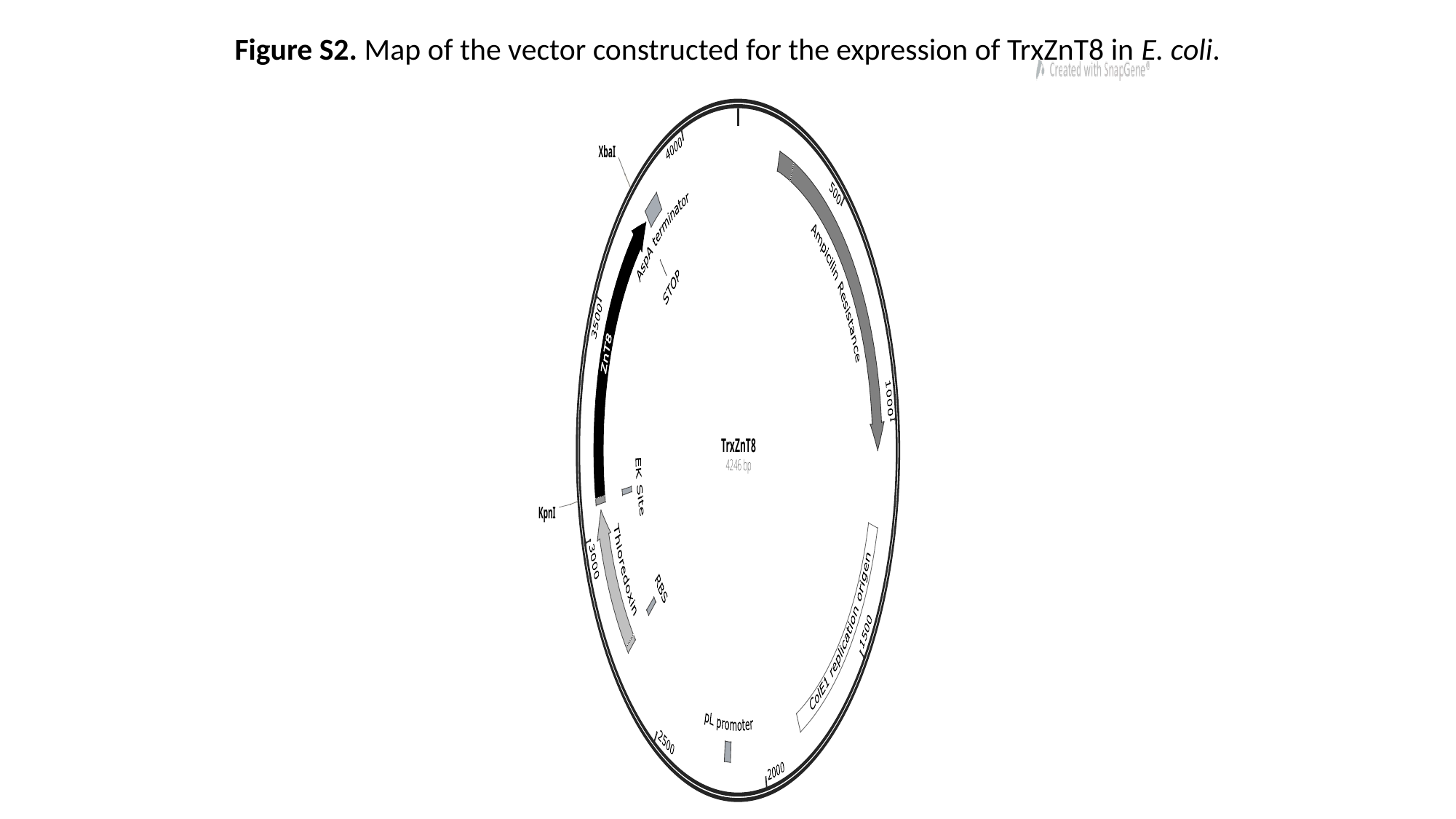

Figure S2. Map of the vector constructed for the expression of TrxZnT8 in E. coli.

Supplement: Supplementary file 3 — Additional file 3: Figure S2. Map of the vector constructed for the expression of TrxZnT8 in E. coli. The ZnT8 sequence was inserted into the multiple cloning site of the expression vector and expressed as an amino terminal fusion to the E. coli protein thioredoxin. To drive expression of thioredoxin fusions, pTrxFus uses the pL promoter from the λ bacteriophage and the AspA transcription terminator. Plasmid selection and maintenance was ensured by the presence of a beta-lactamase gene (BLA) that provide ampicillin resistance. KpnI and XbaI sites are indicated at the 3´ and 5´ ends of the ZnT8 sequence. RBS: ribosome binding site. EK site: enterokinase cleavage site. [file 12934_2017_816_MOESM3_ESM.pptx]
