# Supplementary material for: Prokaryotic expression and characterization of the heterodimeric construction of ZnT8 and its application for autoantibodies detection in diabetes mellitus
Source: Microb Cell Fact. 2017 Nov 13;16:196. doi: 10.1186/s12934-017-0816-4 (PMC5683521; doi:10.1186/s12934-017-0816-4)
Supplement: Supplementary file 2 — Additional file 2: Table S1. Agreement between the pb-ELISA and RBA with sera from type 1 diabetic patients (n = 62) and control individuals (n = 69). The agreement between the two immunoassays was 79.4%, with kappa statistic of 0.54. [file 12934_2017_816_MOESM2_ESM.pdf]

Table S1. Agreement between the pb-ELISA and RBA with sera from type 1 diabetic patients (n=62) and control individuals (n=69)

**pb-ELISA**	**RBA**		Total		Kappa statistic*
	Positive	Negative			
Positive	29	5	34	Po= 0.794<br>Pe= 0.553	0.539
Negative	22	75	97		
Total	51	80	131		

Po: observed agreement, Pe: expected agreement

*A kappa statistic of ≥ 0.75 represents excellent agreement, 0.40 to 0.75 represents good to fair agreement, and < 0.40 represents poor agreement [37, 38].
